# Bisulphide of Carbon in Pulmonary Affections

**Published:** 1886-12

**Authors:** Jainn Guerra Estapè, F. R. Campbell


### *BISULPHIDE OF CARBON IN PULMONARY AFFECTIONS.*

By DR. JAINN GUERRA ESTAPÉ.

Translated from *Revist de Ciencias Medicas* by F. R. Campbell, M. D.

The researches of Peligot and the observations of Dujardin-Beaumetz have placed the action of bisulphide of carbon as an anti-ferment beyond doubt. Having read in a recent work by Dujardin-Beaumetz of the value of bisulphide of carbon for intestinal antisepsis, I experimented upon myself with the compound and found that it was largely eliminated by the respiratory mucus membrane. My breath gave a marked reaction with Fehling's test. This may be tested by blowing the breath through a tube into a cup containing the solution when a blackish, flocculent precipitate of sulphide of copper will be formed if carbon bisulphide is present in the expired air. In this manner I was enabled to establish the harmlessness of the drug when taken in such doses and under such conditions as I will hereafter describe. These experiments served later as a basis for a therapeutic application of bisulphide of carbon.

Three months ago a man forty-eight years of age, a wood-worker, consulted me. His wife informed me the day before the consultation that many physicians in his native place and some in Barcelona had assured her that her husband was suffering with phthisis, founding this diagnosis on a careful physical examination. She told me that his illness was of some four years duration and thought that it arose from a severe cold contracted while at his work. In view of the facts obtained I made a diagnosis of bronchiectasis. I will not describe his symptoms in detail because he presented a typical case of the disease, one which could not be confounded with pulmonary tuberculosis. The heart was normal with the exception of a slight hypertrophy of the left heart. I decided to employ the *aqua sulpho-carbonata* recommended by Dujardin-Beaumetz for intestinal antisepsis, feeling assured from my previous experiments that it would exert a favorable influence upon the fetid purulent excretions. I directed him to pour into a pint bottle the following mixture:

| | | | |
|---|---|---|---|
| ℞ | Aquæ, - - - - - | grams | 500. |
| | Carbon Bisulphid. - - - | " | 25. |
| | Essence Menth pip. - - - | gtt. | xxx. |

This is to be well shaken, then allow the flask to stand a few minutes. Of this solution a tablespoonful in milk should be taken six times a day. I prohibited the use of all alcoholic beverages, since it is well known that alcohol acts upon the carbon bisulphide in the blood, forming sulphuretted hydrogen.

In six days my patient returned and I was not a little surprised to learn that he was no longer troubled with morning cough and that the expectoration had diminished while the fetor had disappeared. The medicine was continued eight days more in the same form and dose, at which time the improvement was so marked that it was perceptible on percussion and auscultation. This treatment combined with the use of tonics, always excluding alcohol, was continued two months when the patient had improved so materially that he returned to his work.

Since treating the above patient I have used carbon bisulphide in many cases of chronic bronchitis, and one of excessive bronchorrhœa and have always obtained the same excellent result.

I have no doubt that my observations are not sufficiently numerous for the deduction of precise conclusions. Yet I hope that my success with this harmless remedy may induce others to test it in some pulmonary affections.

